# Cardiovascular disease risk in women with migraine

**DOI:** 10.1186/1129-2377-14-75

**Published:** 2013-09-06

**Authors:** Fernanda Camboim Rockett, Alexandre da Silveira Perla, Ingrid D Schweigert Perry, Márcia L Fagundes Chaves

**Affiliations:** 1 Postgraduate Program in Medicine: Medical Sciences, Universidade Federal do Rio Grande do Sul, Porto Alegre, Brazil; 2 Food and Nutrition Research Centre - Hospital de Clínicas de Porto Alegre/Universidade Federal do Rio Grande do Sul, Porto Alegre, Brazil; 3 Neurology Service - Hospital de Clínicas de Porto Alegre, Porto Alegre, Brazil; 4 Department of Internal Medicine, Universidade Federal do Rio Grande do Sul, Porto Alegre, Brazil

**Keywords:** Migraine, Cardiovascular disease, Cardiovascular risk, C-reactive protein, Depression, Sleep disturbance

## Abstract

**Background:**

Studies suggest a higher prevalence of unfavourable cardiovascular risk factors amongst migraineurs, but results have been conflicting. The aim of this study was to investigate traditional and newly recognized risk factors as well as other surrogate markers of cardiovascular risk in obese and normal weight women with migraine.

**Methods:**

Fifty-nine adult female probands participated in this case–control study. The sample was divided into normal weight and obese migraineurs and age- and body mass index-matched control groups. The following cardiovascular risk factors were analyzed: serum levels of lipids, fasting glucose, and insulin; insulin resistance; blood pressure; smoking (categorized as current, past or never); Framingham 10-year risk of general cardiovascular disease score; C-reactive protein; family history of cardiovascular disease; physical activity; sleep disturbances; depression; and bioelectrical impedance phase angle. The means of continuous variables were compared using Student’s *t-*test for independent samples or the Mann–Whitney *U-*test (for 2 groups) and ANOVA or the Kruskal-Wallis test (for 4 groups) depending on the distribution of data.

**Results:**

All migraineurs were sedentary irrespective of nutritional status. Migraineurs had higher depression scores and shorter sleep duration, and obese migraineurs, in particular, had worse sleep quality scores. Insulin resistance and insulinaemia were associated with obesity, and obese migraineurs had lower HDL-c than normal weight controls and migraineurs. Also, the Framingham risk score was higher in obese migraineurs.

**Conclusion:**

These findings suggest that female migraineurs experience marked inactivity, depression, and some sleep disturbance, that higher insulin resistance and insulinaemia are related to obesity, and that obesity and migraine probably exert overlapping effects on HDL-c levels and Framingham 10-year cardiovascular risk.

## Background

Migraine is a chronic disorder that affects a large proportion of the population, with a female-to-male ratio ranging from 2:1 to 3:1. This condition predominantly affects middle-aged women and has a complex pathophysiology involving both neuronal and vascular mechanisms [1-4]. Migraine is often characterized by recurrent attacks of moderate to severe pulsating headache, which may be localised in the frontotemporal, unilateral or bilateral areas. The attacks are usually accompanied by nausea, vomiting, and photo-, phono- or osmophobia, lasting 4 to 72 hours, whether or not preceded by focal neurological phenomena known as the migraine aura [5].

Relationships between migraine and classical cardiovascular (CV) risk factors have been suggested in previous studies [3,6,7], but controversy remains [8,9]. Furthermore, no universally accepted mechanism to explain this connection has been put forward so far [10]. In Brazil, there are scarce data on the prevalence of risk factors for cardiovascular disease (CVD) among female migraineurs, especially middle-aged women [10]. The prevalence of obesity among migraineurs is high [11], but little is known about whether this could be a determining factor. Recent data have suggested that normal weight migraineurs exhibit an atherogenic lipid profile, which shares several features with obesity-related lipid alterations [12].

In addition to research into traditional CV risk factors, such as blood pressure (BP), lipid profile, diabetes, and physical inactivity, the study of newly recognized CV risk factors, in particular those related to inflammation [13], oxidative stress [12], and proposed surrogate predictors of CV risk [14-16], could help clarify these unsolved questions. Therefore, hypothesising that migraine would be associated with worse CV risk regardless of nutritional status, the aim of this study was to investigate traditional and newly recognized risk factors as well as other surrogate markers of CV risk in obese and normal weight women with migraine compared to obese and normal weight controls.

## Methods

### Study design and participants

This case–control study used a convenience sampling strategy. Female migraineurs aged 18 to 55 years, treated for at least one year at the outpatient Headache Clinic of the Neurology Service at Hospital de Clínicas de Porto Alegre (HCPA), a tertiary referral hospital in Rio Grande do Sul, Brazil, were recruited between October 2012 and January 2013. All were diagnosed with migraine by a consultant headache specialist according to International Headache Society criteria [5]. Healthy controls matched by body mass index (BMI) and age were recruited voluntarily. According to BMI, subjects were allocated to obese or normal weight groups (obese migraineurs, normal weight migraineurs, obese controls, and normal weight controls). Illiterate subjects, subjects diagnosed with mental retardation or currently taking statins, corticosteroids, or hypoglycaemic and hypolipidaemic agents were excluded from the study.

Socio-demographic data (age, marital status, educational achievement, self-reported skin colour, and socioeconomic status) and anthropometric measurements were collected during an interview and physical examination at the HCPA Clinical Research Centre.

Socioeconomic status was defined on the basis of the Brazilian Association of Research Companies Economic Classification Criterion, which uses purchasing power to stratify the population into five socioeconomic classes, A through E, with “A” representing the richest stratum of society and “E” the poorest [17].

### Migraine features

The following migraine features were assessed: classification; attack frequency, intensity, disability, and duration; family history; and medications used. The severity and disability caused by attacks were assessed on a visual analogue pain scale and by means of the Migraine Disability Assessment Test – MIDAS criteria [18].

### Body measurements

Height was measured with a wall-mounted stadiometer (Harpenden, Holtain®, Crymych, UK) to the nearest 0.1 cm and weight was measured using a digital platform scale with a resolution of 0.1 kg (Toledo®, Model 2096PP/2, São Paulo, Brazil), while subjects were barefoot and wearing lightweight clothing. Weight and height were used to calculate BMI, using the formula BMI=(weight[kg]/height[m]^2^), and nutritional status was classified on the basis of World Health Organization (WHO) cut-off points for adults (18.5-24.9 kg/m^2^ for normal weight and ≥30.0 kg/m^2^ for obese) [19]. Waist circumference (WC) was measured at the narrowest point of the trunk (Sanny Medical®, Model SN-4011, São Paulo, Brazil) and classified according to WHO standards [20], with WC ≥80 or ≥88 cm representing increased or substantially increased risk of cardiovascular disease and metabolic complications, respectively. Body composition (fat and lean mass) was estimated by the bioelectrical impedance analysis (BIA) method with a Biodynamics 450 analyzer (Biodynamics 450® version 5.1, Biodynamics Corporation, Seattle, WA, USA). Resting ECG tab electrodes (Conmed Corporation, Utica, NY, USA) were used. Procedures were carried out to ensure valid BIA measurements as described by Kyle et al. [21,22]. All measures were obtained by a trained nutritionist.

### Cardiovascular disease risk factors

The following risk factors were analyzed: serum levels of lipids, fasting glucose, and insulin; insulin resistance; BP; smoking (categorized as current, past or never); Framingham risk; C-reactive protein (CRP); family history of CVD; physical activity; sleep disturbances; depression; and BIA phase angle (PA).

#### Biomarkers

Blood collection was performed after an overnight fast by venous puncture, immediately centrifuged at 4°C temperature, and serum aliquots were stored in duplicate at −80°C until analysis. All probands were free of common infectious diseases and, in migraineurs, a headache-free period. Lipid profile [triglycerides, total cholesterol, HDL cholesterol (HDL-c, determined using an enzymatic colorimetric method)], plasma glucose (determined by the hexokinase method, Advia 1800), insulin, and CRP were measured. All tests were conducted at the HCPA Pathology Laboratory using standardized methods. LDL-cholesterol (LDL-c) was determined according to the Friedewald equation [23]. Insulin resistance was evaluated by the homeostasis model assessment (HOMA-IR). Oxidized LDL was determined by commercial ELISA (Mercodia, Uppsala, Sweden).

#### Blood pressure

BP was measured using the auscultatory method (Premium® stethoscope, Ningbo Yinzhou Wuhai Medical Instruments Factory, China, and ML035 mercury sphygmomanometer, Solidor, Wenzhou Qianlong Medical Appliance Factory, China). Two measurements were obtained with a 5-min interval, with participants seated, arm level with the heart, and at least 1 hour after the last caffeine and/or tobacco intake. The first measurement was obtained after a 5-min rest, and the mean of the two measurements taken into account for analysis. BP data were analyzed according to the Seventh Report of the Joint National Committee on Prevention, Detection, Evaluation, and Treatment of High Blood Pressure criteria [24]. Participants who had a diastolic BP ≥90 mmHg and/or a systolic BP ≥140 mmHg or reported a prior medical diagnosis of hypertension or were receiving treatment for hypertension (regardless of BP levels) were classified as having hypertension.

#### Framingham risk

To assess CV risk, we calculated the Framingham 10-year risk of general CVD score, which estimates the risk attributable to collected variables (age, sex, total cholesterol, HDL-c, smoking status, and systolic BP stratified for the use of anti-hypertensive medication) [25]. The resulting risk score was examined in quartiles, with the lowest quartile defined as the reference group (score ≤0.6%).

#### Physical activity

Physical activity level was assessed by the International Physical Activity Questionnaire (IPAQ), in its short version, validated in Brazil by Matsudo et al. [26]. According to their individual activity over the days of the week, subjects were classified as sedentary or active.

#### Depression symptoms

To identify and quantify depression symptoms, subjects completed the Beck Depression Inventory (BDI), as validated in Brazilian Portuguese. The BDI consists of 21 statements, the responses to which are scored from 0 to 3. Scores below 10 represent absence of depression; 10–29, mild to moderate depression; and over 30, severe depression [27,28].

#### Sleep quality

The Pittsburgh Sleep Quality Index (PSQI) evaluates sleep quality over the last month, including qualitative and quantitative questions. Using a cut-off score of ≥ 6, as recommended in the original study [29], subjects were classified as having good or poor sleep quality. Sleep duration, derived from the PSQI, was also analyzed and defined as short (≤ 5 hours) or long (≥ 8 hours) [30].

#### Bioelectrical impedance phase angle

Because a low PA has been suggested as a surrogate CV risk factor [14], this parameter was also assessed in our study. Briefly, PA is a parameter obtained from BIA, being a derived measure from the relationship between two electrical properties of tissues, resistance (R) and reactance (Xc), and is determined by the equation [PA°=(Xc/R) × (180°/π)]. It thus describes the phase shift between the current and voltage that results from the electrochemical membrane. A low PA suggests cell death or decreased cell integrity [31].

### Statistical analysis

Data entry was carried out in duplicate. Validation procedures were used to identify and correct data entry errors. Categorical variables were expressed as absolute and relative frequencies, and continuous variables, as mean ± standard deviation or median and interquartile range as appropriate. The chi-square test was used to test for an association between categorical variables. The means of continuous variables were compared using Student’s *t-*test for independent samples or the Mann–Whitney *U-*test (for 2 groups) and ANOVA or the Kruskal–Wallis test (for 4 groups) depending on the distribution of data. Analyses were performed using the Statistical Package for the Social Sciences (SPSS, Chicago, IL, USA), version 18.0, and results were considered significant when p ≤ 0.05.

### Ethical considerations

This study was approved by the HCPA Research Ethics Committee with protocol #12-0215 and was conducted in compliance with international and national guidelines for human subject research. All participants provided written informed consent.

## Results

A total of 59 probands were included in the study. Fifteen normal weight (mean BMI 22.8±1.8 kg/m^2^) and 15 obese (mean BMI 33.3±3.0 kg/m^2^) migraineurs were investigated. The control groups included 15 normal weight (mean BMI 22.2±1.7 kg/m^2^) and 14 obese (mean BMI 34.7±4.6 kg/m^2^) subjects. The socio-demographic characteristics of the sample are given in Table 1. Migraine with aura was diagnosed in six patients; other migraine characteristics are described in Table 2.

**Table 1:** Socio-demographic profile of the sample

**Characteristics**	**Total (n=59)**	**Migraineurs (n=30)**	**Controls (n=29)**	**p-value#**
Economic class (ABEP classification)^a^				
*A*	7 (11.9)	1 (3.3)	6 (20.7)	0.052
*B*	31 (52.5)	15 (50.0)	16 (55.2)	
*C*	21 (35.6)	14 (46.7)	7 (24.1)	
Skin colour (self-reported)				
*White*	47 (79.7)	23 (76.7)	24 (82.8)	0.188
*Black*	6 (10.1)	2 (6.7)	4 (13.8)	
*Other*	6 (10.1)	5 (16.7)	1 (3.4)	
Years of formal schooling				
*<8*	9 (15.3)	8 (26.7)*	1 (3.4)	**0.046**
*8 to 11*	34 (57.6)	15 (50.0)	19 (65.5)	
*>11*	16 (27.1)	7 (23.3)	9 (31.0)	
Marital status				
*Single*	23 (39.0)	9 (30.0)	14 (48.3)	0.321
*Married/Stable relationship*	30 (50.8)	17 (56.7)	13 (44.8)	
*Divorced*	6 (10.2)	4 (13.3)	2 (6.9)	

Categorical variables expressed as n (%).

^a^ABEP, *Associação Brasileira de Empresas de Pesquisa* (Brazilian Association of Research Companies) [17].

#p-value represents comparison between controls and migraineurs (chi-square test). Bold type denotes significant values (p≤0.05).

*(asterisk) indicates the association found.

**Table 2:** Migraine features in normal weight and obese migraineurs

**Characteristics**	**Overall sample (n=30)**	**Normal weight migraineurs (n=15)**	**Obese migraineurs (n=15)**	**p-value#**
Symptoms of aura (%)	6 (20.0)	2 (13.3)	4 (26.7)	0.651
Frequency of attacks (past 3 months)		9.0 (3.0-24.0)	7.0 (3.0-24.0)	
Duration of crisis (hours)		24.0 (3.0-24.0)	24.0 (2.0-72.0)	
Disability (MIDAS grade^a^)				
*I*	11 (36.7)	7 (46.7)	4 (26.7)	0.168
*II*	6 (20.0)	3 (20.0)	3 (20.0)	
*III*	6 (20.0)	4 (26.7)	2 (13.3)	
*IV*	7 (23.3)	1 (6.7)	6 (40.0)	
Severity (visual analogue scale)				
*0 to 4*	2 (6.7)	1 (6.7)	1 (6.7)	0.921
*5 to 7*	9 (30.0)	5 (33.3)	4 (26.7)	
*8 to 10*	19 (63.3)	9 (60.0)	10 (66.7)	
Migraine prophylaxis	15 (50.0)	6 (40.0)	9 (60.0)	0.273
Positive family history of migraine	19 (63.3)	11 (73.3)	8 (53.3)	0.256

Continuous variables expressed as median and interquartile range (P25-P75) and categorical variables as n (%).

^a^MIDAS: Migraine Disability Assessment Test [18].

#p-value represents comparison between migraineurs groups with the chi-square test.

Anthropometric data and CV risk factors are shown in Table 3, stratified by headache status.

**Table 3:** Characteristics and cardiovascular risk factors of migraine patients and controls (n=59)

**Characteristics**	**Normal weight controls (n=15)**	**Normal weight migraineurs (n=15)**	**Obese controls (n=14)**	**Obese migraineurs (n=15)**	**p-value#**
Age (years)	34.0±10.6	34.1±10.6	39.4±9.5	40.4±9.5	0.178
BMI (kg/m^2^)	22.2±1.7^a^	22.8±1.8^a^	34.7±4.6^b^	33.3±3.0^b^	< 0.001
Waist circumference (cm)	70.0±6.2^a^	73.2±6.6^a^	94.5±9.3^b^	97.0±6.7^b^	< 0.001
*<80*	14 (93.3)*	13 (86.7)*	2 (14.3)	0	< 0.001
*≥80 to 87*	1 (6.7)	2 (13.3)	2 (14.3)	3 (20.0)	
*≥88*	0	0	10 (71.4)*	12 (80.0)*	
Phase angle (°)	6.5±0.5	6.5±0.8	6.6±0.8	6.8±0.7	0.669
Body fat (%)	25.7±3.0^a^	27.7±4.0^a^	38.4±3.3^b^	37.2±3.7^b^	< 0.001
Body mass, lean (%)	74.2±3.0^a^	72.2±4.0^a^	61.5±3.3^b^	62.7±3.7^b^	< 0.001
Total cholesterol (mg/dL)	192.0±34.9	185.6±27.2	188.0±32.1	185.6±30.9	0.937
HDL-c (mg/dL)	54.9±11.9^a^	54.8±13.3^a^	46.7±14.0^a.b^	42.5±11.5^b^	0.022
LDL-c (mg/dL)	115.0±27.1	112.9±20.3	111.2±32.9	112.9±24.0	0.985
LDL-c, oxidized (U/L)	75.7±21.0	67.8±22.5	69.3±18.9	86.6±8.9	0.069
Triglycerides (mg/dL)	81.0 (60.0-123.0)	79.0 (57.0-120.0)	141.0 (86.2-194.7)	103.0 (79.0-223.0)	0.333
Fasting glucose (mg/dL)	81.0 (78.0-87.0)	82.0 (78.0-86.0)	86.0 (80.0-103.7)	89.0 (81.0-96.0)	0.086
Insulin, serum (μUI/mL)	7.8 (6.4-12.8)^a^	5.7 (4.6-10.1)^a^	17.7 (13.5-17.7)^b^	14.6 (11.8-22.2)^b^	< 0.001
HOMA-IR	1.5 (1.3-2.6)^a^	1.2 (0.9-2.0)^a^	3.6 (2.7-5.0)^b^	3.2 (2.2-4.3)^b^	< 0.001
Systolic BP (mmHg)	112.5 (102.5-120.0)^a.b^	115.0 (110.0-120.0)^a^	120.0 (118.7-126.2)^a.b^	122.5 (110.0-130.0)^b^	0.013
Diastolic BP (mmHg)	70.0 (70.0-70.0)^a.b^	72.5 (70.0-87.5)^a^	80.0 (80.0-85.0)^b^	80.0 (70.0-90.0)^b^	0.003
CRP (mg/L)	4.0 (4.0-4.0)	4.0 (4.0-4.0)	10.0 (4.0-12.4)	4.0 (4.0-18.1)	0.057
Framingham risk score					
*≤0.6*	7 (46.7)*	6 (40.0)	1 (7.1)	1 (6.7)	0.015
*>0.6*	8 (53.3)	9 (60.0)	13 (92.9)	14 (93.3)*	
Cardiovascular disease in 1^st^ degree relative					
*Yes*	5 (33.3)	5 (33.3)	6 (42.8)	1 (6.7)	0.156
*No*	10 (66.7)	10 (66.7)	8 (57.1)	14 (93.3)	
Smoking status					
*Current*	1 (6.7)	2 (13.3)	3 (21.4)	1 (6.7)	0.685
*Past*	3 (20.0)	4 (26.7)	4 (28.6)	2 (13.3)	
*Never*	11 (73.3)	9 (60.0)	7 (50.0)	12 (80.0)	
Physical activity (IPAQ)					
*Sedentary*	9 (60.0)	15 (100.0)	14 (100.0)	15 (100.0)	**< 0.001**
*Active*	6 (40.0)*	0	0	0	
Depression (BDI)					
*Absence*	15 (100.0)*	6 (40.0)	9 (64.3)	7 (46.7)	**0.012**
*Mild/moderate*	0	8 (53.4)*	4 (28.6)	5 (33.3)	
*Severe*	0	1 (6.6)	1 (7.1)	3 (20.0)*	
Sleep duration (h)					
*≤ 5*	0	5 (33.3)	3 (21.4)	5 (33.4)	0.092
*6 to 8*	14 (93.3)	10 (66.7)	9 (64.3)	7 (46.6)	
*> 8*	1 (6.7)	0	2 (14.3)	3 (20.0)	
Sleep quality (PSQI)	6.2±2.7^a.b^	7.2±3.5^a.b^	5.0±3.8^a^	9.6±5.0^b^	**0.017**
*Good*	6 (40.0)	5 (33.3)	9 (64.3)	4 (26.7)	0.188
*Poor*	9 (60.0)	10 (66.7)	5 (35.7)	11 (73.3)	

*BDI* Beck Depression Inventory, *BMI* body mass index, *BP* blood pressure, *CRP* C-reactive protein, *HDL-c* high-density lipoprotein cholesterol, *HOMA-IR* Insulin Resistance Homeostasis Model Assessment, *IPAQ* International Physical Activity Questionnaire, *LDL-c* low density lipoprotein cholesterol, *PSQI* Pittsburgh Sleep Quality Index, *WC* waist circumference; Framingham score – General cardiovascular disease (10-year risk) [25]. Lowest quartile used as reference category (≤0.6%).

Continuous variables expressed as mean ± standard deviation or median and interquartile range, and categorical variables, as n (%).

#p-value represents comparison among four groups with one-way ANOVA or Kruskal–Wallis test. Bold type denotes significant values (p≤0.05).

Different superscript letters represent averages/medians significantly different between groups.

*(asterisk) indicates the association found.

There were no significant between-group differences in PA, nor in the following traditional CV risk factors: total cholesterol, LDL-c, triglycerides, and glucose. However, obese migraineurs had lower HDL-c compared to normal weight groups (controls and migraineurs), although no difference was found compared to obese controls. Insulin resistance (measured by HOMA-IR) and insulinaemia were associated with obesity. When compared to normal weight migraineurs, obese migraineurs had significantly higher mean systolic BP values. Diastolic BP was lower in normal weight migraineurs than in obese controls and obese migraineurs. Although the difference was not significant, obese migraineurs tended to have higher levels of oxidized LDL. Most of the study subjects were non-smokers (n=52; 88.1%), and this risk factor was not associated with any group. All migraineurs were sedentary irrespective of nutritional status, whereas normal weight controls were physically active. A history of CVD in first-degree relatives was reported with equal frequency by all groups.

Framingham risk score was higher in obese migraineurs, whereas normal weight controls had lower scores.

Levels of CRP, a newly recognized CV risk factor related to inflammation, followed a broadly similar pattern across all groups.

Regarding depression, migraineurs had higher BDI scores irrespective of nutritional status, consistent with mild/moderate depression in normal weight migraineurs and severe depression in obese migraineurs. In controls, normal weight was associated with absence of depression.

There were no differences in sleep duration between the study groups. When migraineurs and controls were compared independently of BMI, migraineurs were more likely to experience short sleep duration (p=0.033; chi-square test). Headache and nutritional status were not associated with sleep quality as assessed by PSQI score. Pooled analysis of all migraineurs revealed the same profile (p=0.089; chi-square test). However, the mean scores of obese migraineurs were higher than those of obese controls. This was also evident on comparison between all migraineurs and all controls, regardless of nutritional status (8.4±4.3 and 5.6±3.3 for migraineurs and controls respectively; p=0.009; Student *t*-test).

## Discussion

In the present study, we investigated traditional CV risk factors (lipid profile, abnormalities in serum glucose and insulin levels, oxidative stress, BP, physical activity, and smoking), a newly recognized CV risk marker (CRP), and a series of surrogate factors (sleep quality and duration, depression, and bioelectrical PA) in obese and normal weight migraineurs and age- and BMI-matched controls, hypothesising that migraine would be associated with worse CV risk, regardless of nutritional status. Our main findings were that (a) obese migraineurs had lower HDL-c than normal weight controls and migraineurs, (b) insulin resistance and insulinaemia were associated with obesity, (c) all migraineurs were sedentary irrespective of nutritional status, (d) Framingham risk scores were higher in obese migraineurs, (e) migraineurs had higher depression scores and shorter sleep duration, and (f) migraineurs, both obese and overall, had worse sleep quality scores.

In this study, obese migraineurs exhibited higher CV risk (as measured by Framingham 10-year risk for general CVD scores) than normal weight migraineurs or controls (the latter regardless of nutritional status), whereas normal weight controls seemed to be protected against an unfavourable risk profile. Nevertheless, there seems to be an overlap of migraine and obesity in terms of CV risk profile in migraineurs. Some recent studies have reported on CV risk profiles in this population [6,8,31,32]. Winsvold et al. [33] suggest that a possible mechanism linking headache to CV risk profile is that headache, especially if frequent, may lead to lifestyle changes such as decreased physical activity, altered smoking habit, or unhealthy diet. Stam et al. [34] quantified atherosclerosis by intima media thickness, pulse wave velocity, and ankle-brachial index and found no differences between migraineurs and controls, concluding that enhanced atherosclerosis is an unlikely explanation for CV risk in migraine patients.

It bears noting that none of the migraineurs in our sample reported physical activity, whereas normal weight controls clearly led an active life. It is well known that higher levels of physical activity are associated with fewer CV events. Although the precise mechanisms underlying this inverse association are unclear, differences in several CV risk factors may mediate these effects, particularly inflammatory/haemostatic factors and BP [35]. It is also widely established that exercise is reported by migraineurs as a trigger of attacks, which may explain why some patients with migraine avoid physical activity [36]. In our study, underlying factors associated with physical activity, such as insulinaemia, insulin resistance, and HDL-c, were only related to obesity, and BP values remained within the reference range in all groups [35]. Therefore, headache status and the associated physical inactivity do not appear to be the link that connects these factors. Nevertheless, our findings corroborate those of an earlier study showing that migraineurs engage in less physical activity than individuals without headache [37].

Conversely, considering the component items of the Framingham score, there was an insignificant number of smokers among obese migraineurs and control groups, and cholesterol profile and diabetes status did not differ either by headache status or by nutritional status. However, HDL-c was lower in obese migraineurs than in all other groups and systolic BP was higher compared to normal weight migraineurs, although remaining, as mentioned above, within the clinically normal range. Furthermore, other traditional CV risk factors, such as triglycerides, glucose, HOMA-IR index, LDL-c, and insulinaemia, did not differ among groups. Differences in systolic and diastolic BP values, despite being statistically significant, were not clinically relevant. These data suggest that isolated factors do not play a significant role in determining risk, but combinations of risk factors may have been decisive determinants of CV risk in obese migraineurs as evaluated by the Framingham score. It bears noting that a previous longitudinal Brazilian study also failed to find any differences in BP, diabetes prevalence, and total serum cholesterol levels between elderly migraineurs and controls, differing from our data with regard to HDL-c and Framingham risk scores. Overall, the authors did not find a worse CV profile among migraineurs using the Framingham 10-year risk of myocardial infarction or coronary heart disease death scores [10]. It is also important to note that we evaluated general CV risk by Framingham score, while several previous studies have examined risk of stroke [38,39], coronary heart disease [7,9], myocardial infarction [33] or first coronary events [32], limiting the strength of comparisons.

De Luis et al. [14] reported differences in fat mass, glucose, HOMA-IR and inflammatory markers such as leptin and IL-6 levels in different bioelectrical PAs in obese women, suggesting that a low tertile of PA could be a surrogate CV factor. However, the biological meaning of PA has yet to be fully understood. A lower PA suggests cell death or decreased cell integrity, whereas a higher PA suggests large quantities of intact cell membranes [40]. Despite its association with worse prognosis in established chronic heart failure [31], the role of PA as a predictor of CV risk in subjects without baseline CVD is virtually nonexistent, except for the above-cited study. In our investigation, neither headache nor nutritional status showed any relation to PA. We also found no difference among groups in the factors mentioned by De Luis et al. [14] as associated with variation in PA, such as glucose, HOMA index, and CRP levels. To our knowledge, the present study was the first to attempt to quantify PA in migraineurs. Further studies are required to ascertain whether PA is affected by migraine and whether the hypothesised use of PA as a surrogate marker of CV risk in obese women [14] also applies to non-obese women.

CRP is a sensitive indicator of active systemic inflammation and an independent risk marker for CV morbidity, including ischaemic stroke [41]. A small, uncontrolled retrospective review found abnormal CRP levels in migraineurs with complex clinical features [42]. Although no significant differences have been seen in obese or normal weight migraineurs compared with controls [12], several studies support the role of inflammation in migraine [43,44]. It is also noteworthy that, although the difference was not significant, obese migraineurs tended to have higher levels of oxidized LDL. Our results, derived from a small sample, need confirmation in larger controlled studies. Furthermore, prospective studies may establish whether measurements of CRP and oxidative parameters can identify patients with migraine at risk of CV events.

Few observational studies have reported a positive association between depression and incidence of coronary heart disease [45]. In a large prospective community-based cohort, depressive symptoms at baseline were consistently associated with fatal coronary heart disease and stroke events [46]. In another cohort of middle-aged women without baseline coronary heart disease, depressive symptoms were associated with higher risks of cardiac events [15]. Part of this association could be explained by increased risk of fatal ventricular arrhythmias [15]. Additionally, a relationship between migraine and depression has been proved by several studies [47-49], and is reported to be bidirectional [47,50]. Our data corroborate these associations, with migraineurs exhibiting mild/moderate and severe depression. Recurrently, obese migraineurs showed the worst profile, suggesting overlapping effects of obesity and headache.

There is emerging evidence that sleep parameters, specifically sleep duration [30] and quality of sleep [16], are associated with CV outcomes. Studies point to a U-shaped association between sleep duration and coronary heart disease and stroke [30]. The prevalence of sleep abnormalities has been reported as being much higher in migraineurs as compared with controls [51,52]. In our study, even though no between-group differences in sleep quality were found, we detected shorter sleep duration in migraineurs compared to controls, regardless of nutritional status. This could affect migraine severity [53] and headache threshold [54] and be associated with worse CV outcomes, such as greater risk of developing or dying of coronary heart disease and stroke, as described by Cappuccio et al. [30] in a meta-analysis of prospective studies. Nevertheless, the known CV outcomes correlated with sleep disorders, such as hypertension [55], diabetes [56], dyslipidaemia, and hypercholesterolaemia [57], were not detected in our study. Obesity itself did not show any influence on these parameters. Likewise, sleep quality was not associated with any of the study groups.

Although Bigal et al. [32] described, in a large population-based study, that both migraine with and without aura were associated with risk factors for CVD, several studies point to a much stronger association for migraine with aura [9,58,59]. Stratification by migraine subtype could shed some light on this relationship, but this was not possible in our sample, because only six patients were diagnosed with migraine with aura.

Further studies with larger sample sizes as well as prospective controlled studies investigating additional recognized biomarkers, such as oxidized LDL-c, lipoprotein A, other inflammatory markers, and eating behaviours, may help ascertain whether migraine is associated with unfavourable CV risk and whether these features are independent of obesity acting as a potentiating factor.

Some strengths of this study are that diagnosis of migraine was made by a headache specialist, interviews were conducted and anthropometric parameters were measured by a trained nutritionist, and several surrogate markers were evaluated in addition to conventional parameters. A limitation is the small sample size and the convenience sampling strategy employed, which precluded further or more robust statistical analyses; therefore, we cannot make assumptions about the directionality of the relationship between migraine and CV risk factors or extrapolate results to the overall population of migraineurs.

## Conclusions

Our findings point to marked inactivity, depression, and some sleep disturbances in female migraineurs, and that the effects of obesity and migraine on HDL-c levels and Framingham 10-year general CV risk probably overlap. Therefore, we believe that clinicians should maintain heightened vigilance for these modifiable CV risk factors in migraineurs. This additional attention is justified by well established benefits of modifying these factors. Also, monitoring BMI and other risk factors for CVD is accepted as good practice in patients with migraine.

## Abbreviations

BDI: Beck depression inventory; BIA: Bioelectrical impedance analysis; BMI: Body mass index; BP: Blood pressure; CRP: C-reactive protein; CV: Cardiovascular; CVD: Cardiovascular disease; HCPA: Hospital de clínicas de porto alegre; HDL-c: HDL cholesterol; HOMA-IR: Homeostasis model assessment – insulin resistance; IPAQ: International physical activity questionnaire; LDL-c: LDL-cholesterol; MIDAS: Migraine disability assessment test; PA: Phase angle; PSQI: Pittsburgh sleep quality index; R: Resistance; WHO: World health organization; Xc: Reactance.

## Competing interests

The authors declare that they have no competing interests.

## Authors’ contributions

FCR participated in the work’s conception; design; data collection, interpretation and analysis; have been involved in drafting the manuscript, revising it critically for important intellectual content; and have given final approval of the version to be published. ASP contributed with the literature review and study design. IDSP participated in the work’s conception; design; data interpretation; have been involved in drafting the manuscript, revising it critically for important intellectual content; and have given final approval of the version to be published. MLFC contributed with study design and manuscript preparation. All authors read and approved the final manuscript.
